# Cardiac Obesity and Cardiac Cachexia: Is There a Pathophysiological Link?

**DOI:** 10.1155/2019/9854085

**Published:** 2019-09-02

**Authors:** K. Selthofer-Relatić, A. Kibel, D. Delić-Brkljačić, I. Bošnjak

**Affiliations:** ^1^Department for Cardiovascular Disease, University Hospital Osijek, Josipa Huttlera 4, 31000 Osijek, Croatia; ^2^Department for Internal Medicine, Faculty of Medicine Osijek, University Josip Juraj Strossmayer Osijek, Josipa Huttlera 4, 31000 Osijek, Croatia; ^3^Department for Physiology and Immunology, Faculty of Medicine Osijek, University Josip Juraj Strossmayer Osijek, Josipa Huttlera 4, 31000 Osijek, Croatia; ^4^Department for Internal Medicine, School of Medicine, University of Zagreb, Šalata 3, 10000 Zagreb, Croatia; ^5^Clinic for Cardiology, University Hospital “Sestre Milosrdnice”, Vinogradska Cesta 29, 10000 Zagreb, Croatia

## Abstract

Obesity is a risk factor for cardiometabolic and vascular diseases like arterial hypertension, diabetes mellitus type 2, dyslipidaemia, and atherosclerosis. A special role in obesity-related syndromes is played by cardiac visceral obesity, which includes epicardial adipose tissue and intramyocardial fat, leading to cardiac steatosis; hypertensive heart disease; atherosclerosis of epicardial coronary artery disease; and ischemic cardiomyopathy, cardiac microcirculatory dysfunction, diabetic cardiomyopathy, and atrial fibrillation. Cardiac expression of these changes in any given patient is unique and multimodal, varying in clinical settings and level of expressed changes, with heart failure development depending on pathophysiological mechanisms with preserved, midrange, or reduced ejection fraction. Progressive heart failure with misbalanced metabolic and catabolic processes will change muscle, bone, and fat mass and function, with possible changes in the cardiac fat state from excessive accumulation to reduction and cardiac cachexia with a worse prognosis. The question we address is whether cardiac obesity or cardiac cachexia is to be more feared.

## 1. Introduction

In recent years, obesity and visceral fat have been recognized as a worldwide health problem and an independent risk factor for metabolic and cardiovascular diseases such as insulin resistance, dyslipidaemia, arterial hypertension, chronic subclinical inflammation, atherosclerosis, and cardiac steatosis. These obesity-related syndromes can lead to diabetes mellitus; hypertensive heart disease; coronary artery disease, and ischemic cardiomyopathy, diabetic cardiomyopathy, metabolic- and obesity-related cardiomyopathy, coronary microcirculatory dysfunction, and atrial fibrillation. Depending on the level and type of obesity, life style, genetic predisposition, gender, ageing, clinical presentation, and treatment, these disorders can lead to heart failure (HF) with preserved, midrange, or reduced ejection fraction [1–5].

Unanswered questions remain regarding the period between the onset of the problem (obesity) until HF and cardiac cachexia (CC) develop; these questions include the etiology of visceral obesity, the process by which healthy fat tissue becomes stressed, the role of genetics, environment, gender, and ageing, mechanical, and metabolic effects of excess adipose visceral tissue on the cardiovascular system, and the role of inflammation and catabolism in heart failure-related cachexia [1].

## 2. Obesity Background

During recent years, great interest in obesity pathophysiology and related morbidities has led to the development of many different terms to describe obesity-related processes. The term obesity refers to an excess of fat tissue in an organism, regardless of type, location, function, and whether it is “healthy” or “sick” fat tissue [6]. In recent years, the term “obesity paradox” has been used to describe the potential role of obesity in cardiac disease, but it has recently been suggested that this term should be abandoned because it remains figurative without a specific definition proven in studies [7]. “Metabolically healthy but obese” individuals are genetically resistant to adverse metabolic consequences associated with excessive subcutaneous body fat and lower visceral fat, while “metabolically obese but normal weight” subjects may have metabolic abnormalities with increased levels of visceral fat and low levels of subcutaneous fat [7–9].

Subcutaneous adipose tissue (SAT) represents 85% of total adipose tissue mass in lean and obese individuals, while 15% constitutes visceral adipose tissue (VAT) at the highest risk for metabolic dysregulation, suggesting quality is more important than quantity [10, 11]. SAT and VAT are different tissues embryological, histologically, and pathophysiologically. The etiopathogenesis of their development is largely unknown, and elucidation of the mechanisms thereof would be crucial for better understanding. It was initially thought VAT accumulation resulted from SAT overaccumulation. This theory, however, does not explain the phenomenon of “metabolically diseased with normal weight” individuals [1, 12]. Other theories for VAT growth have suggested that an increase in body fat results in adipocyte hypertrophy, that additional adipocytes can differentiate and proliferate in visceral compartments, and that visceral organs cannot handle increased levels of triglycerides [13].

Adipose tissue serves as a central nexus of metabolic communication and control, an arbiter of thermoregulation, a buffer against trauma and cold temperatures, a regulator of reproduction, and satiety. The number of adipocytes in a given individual is mainly determined in childhood and adolescence and remains constant during adulthood in both lean and obese subjects. An increase in fat mass in adulthood is primarily attributed to adipocyte hypertrophy or via hyperplasia in response to overfeeding [14, 15]. Adipose tissue is composed of adipose stem cells, adipocytes, and various other cell types including mural, endothelial, and neuronal cells. In the nondiseased obese, SAT and VAT are different in embryogenesis, genetic predisposition, ageing, and imbalance between adipogenesis and adipocyte apoptosis as a result of neural and vascular networking, anatomy, adipocyte histology, physiology, gender differences, clinical effects, and prognostic differences [1, 11, 16–22].

## 3. Cardiac Visceral Adipose Tissue

Cardiac visceral adipose tissue is composed of local visceral pericardial and intracardial visceral fat according to anatomy, local, and systemic activity. Epicardial adipose tissue and intramyocardial fat are the two main compartments of cardiac visceral fat, with cardiac steatosis as a special pathophysiological entity.

*Epicardial adipose tissue* (EAT) is regional visceral fat surrounding the heart in direct contact with the epicardial conductive coronary artery. EAT makes up about 20% of cardiac weight and lies between the heart and the visceral part of the pericardial layer, is derived from the splanchnopleuric mesoderm, and shares the same embryologic origin as mesenteric and omental fat, composed from fat cells, nervous tissue, nodal tissue, inflammatory, stromal, and immune cells [23–26].

EAT has a high rate of free fatty acid (FFA) synthesis, and incorporation and degradation depend upon myocardial need [24]. EAT contains a high number of mature adipocytes, stromal preadipocytes, macrophages, and lymphocytes which are the source of proinflammatory molecules such as IL-1*β*, IL-6, IL-8, IL-10, TNF-*α*, MCP1, and PAI. This fat is also the source of many adipokines with proatherogenic and proinflammatory effects, leading to decreased levels of protective adiponectin [27–30]. Adipokines can enter directly into the lumen of the vasa vasorum and be transported into the arterial wall where they exert an influence on cells in and around atherosclerotic plaque [31, 32]. In obesity, EAT volume increases substantially, and its pathophysiology changes; these changes include loss of its capability for triglyceride storage and increased lipolysis. The tissue becomes hypoxic and dysfunctional indicators of diseased EAT. Macrophages and lymphocytes infiltrate EAT and secrete proinflammatory cytokines contributing to the creation of a suitable environment for the development of atherosclerosis [26, 29, 33].

Coronary artery calcification is a major vascular injury and considered to be the manifestation of coronary atherosclerosis [34]. EAT facilitates coronary artery calcification progression as a result of direct local fat deposits surrounding coronary arteries. Epicardial fat volume is a significant predictor of obstructive coronary artery disease (CAD). EAT can be a useful marker of CAD in asymptomatic patients with noncalcified plaques and zero calcium scores [19, 35, 36]. EAT correlates with CAD independent of other cardiovascular risk factors and accumulation of EAT is implicated in early CAD development [11, 37].

*Cardiac steatosis* is an endogenous source of cytosolic FFAs, typically found in patients with obesity and type 2 diabetes mellitus, and is known to increase with age. Circulating FFAs participate in the regulation of myocardial fat depots [38, 39]. Lipid accumulation in the myocardium is a result of an imbalance between uptake and utilization of FFAs. Excessive uptake of FFAs activates their oxidation and elicits lipotoxicity, causing impaired cardiac function [40, 41]. When the oxidative capacity of myocardial mitochondria is exceeded, cardiac function can be compromised, with greater left ventricle mass and load, suppressed septal wall thickening, and a decline in diastolic function [42, 43]. The possibility of the identification of myocardial triglyceride content in cardiac steatosis is of great importance, as this could be a potential target for left ventricular diastolic dysfunction treatment [44–46]. The ischemic heart uses glucose as a main energy source, leaving triglycerides unoxidized, creating FFA deposits, and leading to myocardial apoptosis and adverse LV remodelling [47–50].

*Intramyocardial fat tissue* has been histologically validated in healthy obese and nonobese individuals as well as in diseased hearts. This tissue is predominantly located in the right ventricle, with very small amounts present in the apical part of the left ventricle, usually as a part of scar connective tissue after myocardial infarction or as a part of dilated cardiomyopathy [10, 51]. No consensus has been reached about the potential endocrinological role of intramyocardial fat in heart disease nor its relation to conductive system disturbances, especially with sudden cardiac death [42, 51–53]. Recent studies have demonstrated that intramyocardial fat contributes to ventricular electrophysiological remodelling in patients with ischemic heart disease and influences endocardial scar mapping during electrophysiological exams of ventricular tachycardia [54, 55].

## 4. Obesity and Ageing

In addition to obesity, ageing also brings with it increased health problems and risk for stroke, heart attack, and diabetes and an increased likelihood of developing abdominal obesity and fat depots in skeletal muscle, which predisposes the body to insulin resistance and metabolic syndrome [56, 57]. Obesity in elderly individuals is a significant concern, and a better understanding of the mechanisms of age-related diseases is imperative [58]. Potential factors in the interaction of obesity and ageing include inflammation in adipose and other ageing tissues, suppressed adiponectin production, leptin production and leptin resistance, reduction in lean mass, upregulation of the p53 oncogene, reduction of growth hormone secretions, late-onset hypogonadism, changes in thyroid and adrenal function, brown adipocyte induction, and adipose tissue expansion. Age-related changes in metabolism and fat distribution in the body may be crucial features of a vicious cycle that could accelerate the onset of age-related diseases [56, 57].

## 5. Obesity-Related Heart Failure

The link between obesity and heart disease is complex. Obesity increases the potential for developing other risk factors for heart disease, triggers inflammatory processes, and leads to structural and functional changes in the heart itself. Metabolic effects of diseased adipose tissue include adipocyte dysfunction and inflammation leading to type 2 diabetes mellitus, arterial hypertension, dyslipidaemia mixta, coronary atherosclerosis, and various etiological and clinical presentations of cardiomyopathy. Mechanical effects of excessive fat include sleep apnoea, thromboembolic events, increased blood volume, and cardiac output, atrial enlargement and ventricular dilation, and electrocardiogram and conduction system abnormalities [1, 11, 16].

Obesity-related factors are implicated in 11% of HF cases in men and 14% in women. Obesity may directly result in HF by inducing hemodynamic and myocardial changes that lead to cardiac dysfunction, or indirectly through an increased predisposition to other heart failure risk factors. Increased metabolic demands resulting from excess adipose tissue and nonfat mass in obesity lead to hyperdynamic circulation and increased blood volume and cardiac output [5]. When obese individuals develop myocardial dysfunction in the absence of other causes of HF, they are considered to have “obesity cardiomyopathy” [59]. Impairment of ventricular systolic function is not consistently present in obese persons, while obesity-related changes to left ventricular (LV) filling pressures may be due to altered loading conditions and/or an increased LV mass and hypertrophy that reduces ventricular compliance [60]. Obesity-related HF with a preserved ejection fraction is an important phenotype prevalent in those with metabolic disorders. These individuals exhibit a marked expansion of plasma volume, most likely as a result of cardiac microvascular rarefaction acting in concert with myocardial and pericardial fibrosis [61]. The relation of obesity and HF to reduced ejection fraction is indirect via atherosclerosis. Key manifestations of atherosclerosis manifest as coronary heart disease, ischemic stroke, peripheral artery disease, and heart failure [62].

## 6. Cardiac Cachexia

Cardiac cachexia is a severe, complex, multifactorial condition associated with chronic heart failure that can occur independent of age, ventricular function, or functional classification. Presence of the syndrome predicts increased morbidity and mortality related to weight loss and systemic inflammation. CC is defined as at least 5% oedema-free body weight loss in the previous 12 months (or a body mass index < 20 kg/m^2^) in patients with chronic illness and at least three of the following clinical or laboratory criteria: decreased muscle strength, fatigue, anorexia, low fat-free mass index, and abnormal biochemistry characterized by increased inflammatory markers. It involves loss of muscle, fat, and bone. Skeletal muscle wasting and loss of function, called sarcopenia, often precedes cachexia and predicts poor outcome [16, 58, 63, 64].

The underlying pathophysiological mechanisms of CC are not fully elucidated, impeding effective treatment strategies. Because it is different from malnutrition or anorexia, both of which can be reversed with adequate nutrition, CC presents unique therapeutic challenges. Various definitions have been used, adding additional criteria on top of the requisite weight loss [16, 65]. Prevalence of the condition is therefore between 8 and 42%, depending on the study and definition used. There is no one specific marker for CC, which is plagued by heterogeneity in diagnosis and monitoring [58]. An ideal biomarker for CC must be well validated, sensitive, specific, low cost, and able to distinguish between cachexia and sarcopenia [65, 66]. The burden of cachexia on patients and the healthcare system is immense, as cachexia significantly prolongs hospitalization duration and leads to significantly higher costs per hospital stay [65].

Known contributing factors to CC development include disruption of the normal functioning of the gastrointestinal tract, food intake reduction, immunological and neurohormonal activation, and an imbalance between anabolic and catabolic processes [16]. Systemic inflammation can accompany CC, with upregulation of circulating proinflammatory cytokines, including TNF-*α*, IL‐1, and IL‐6, as well as a decrease in anti-inflammatory mediators including IL‐10 and TBF*β*1 [65]. Recent studies have associated right ventricular heart failure with CC development as a result of gastrointestinal venous congestion, neurohumoral/inflammatory activation, malabsorption, protein-loss enteropathy, and gut bacteria translocation [67].

Adiponectin levels rise alongside increases in HF severity and are at the highest levels in patients with CC. An association between elevated serum adiponectin and decreased peripheral muscle mass and muscle strength has been found in elderly heart failure patients who are not cachectic and who do not have diabetes mellitus [65].

Experimental animal models have demonstrated that food restriction or cachexia can lead to ultrastructural, morphological, and functional changes even in normal, disease-free hearts, implying that CC might further contribute to cardiac dysfunction and lead to abnormalities in heart structure and function after HF has already been established, potentially triggering a fatal vicious cycle [16]. One of the most clinically relevant features of CC is skeletal muscle loss, as it determines physical capacity and symptomatic severity of heart failure [65].

Healthcare professionals have defined an urgent need to develop effective preventive and therapeutic modalities because of the substantial burden of CC on patients and the healthcare system, but progress remains slow. With so many unknown interacting and complex pathophysiological mechanisms, efficient targeting of specific pathophysiologic processes to achieve desired results remains elusive. No single intervention can achieve desired therapeutic results in this multifactorial process, but research into prevention and therapy has focused on certain possible pathways. Prevention of CC development includes management of heart failure including pharmacological agents such as beta blockers and ACE-inhibitors. Exercise can contribute to the preservation and increase in muscle mass in addition to anabolic effects [65]. Nutritional interventions for CC patients include protein supplementation, caloric supplementation, and chronic oral carnitine administration in HF patients. Nutritional interventions, however, do not affect the underlying catabolic processes of cachexia. Other agents such as eicosapentaenoic acid, *β*‐hydroxy‐*β*‐methylbutyrate, and resveratrol can potentially counteract body wasting, but clear evidence of efficacy in human subjects is yet to reveal itself [64, 65, 68, 69]. Some additional pharmacological agents are studied to treat CC, including anti-inflammatory drugs such as anti‐TNF-*α*, anti‐IL‐1, and anti‐IL‐6 antibodies, thalidomide, and pentoxifylline, appetite stimulants (megestrol acetate, cannabinoids, and ghrelin), anabolic steroids, *β*‐2 receptor agonists, selective androgen receptor modulators, enzyme inhibitors, and statins, among others [65, 70, 71].

## 7. Discussion and Conclusions

**Figure 1:**
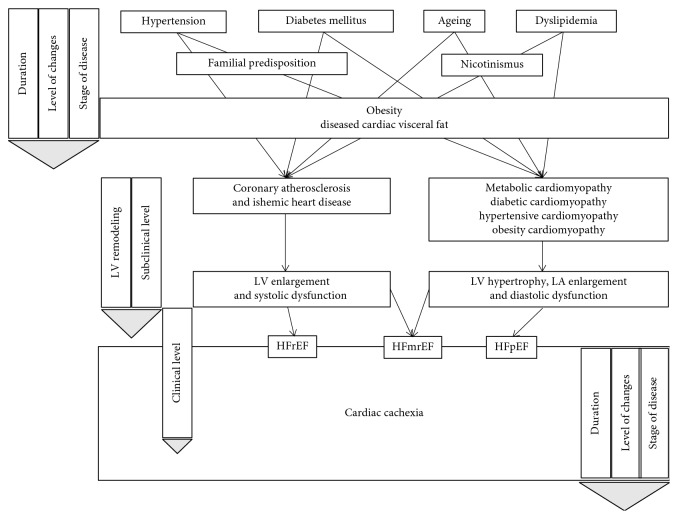
Algorithm of pathophysiological changes of obesity-related heart failure and consequence cardiac cachexia development, from subclinical/predisease level to clinical manifest disease. LA: left atrium; LV: left ventricle; HFrEF: heart failure with reduced ejection fraction; HFmrEF: heart failure with midrange ejection fraction; HFpEF: heart failure with preserved ejection fraction.

Obesity is a known risk factor and a known etiopathogenic factor for a number of cardiovascular and metabolic diseases, including arterial hypertension, diabetes mellitus type 2, dyslipidaemia, and atherosclerosis [1–4]. The exact mechanisms through which obesity exerts its effects on the development of these disorders are complex and multifactorial, despite being of substantial relevance to them. Cardiac visceral obesity may be an important pathogenic factor, including epicardial adipose tissue, perivascular adipose tissue, and intramyocardial fat [11], contributing directly to atherosclerotic coronary disease and ischemic cardiomyopathy with HF development with reduced or midrange ejection fraction, or indirectly to typical metabolic cardiomyopathy with left ventricular hypertrophy, left atrium enlargement, and diastolic dysfunction, and finally HF with preserved ejection fraction (Figure 1) [5, 62]. It is not yet clear to what extent the presence and quantity, much less information about the qualitative composition and function, of cardiac fat regulate the physiological process, nor the extent to which it adversely affects pathophysiological processes.

Individuals with similar body mass index (BMI) may have different metabolic and cardiovascular risk profiles. Tendency to cardiometabolic complications associated with obesity is not solely mediated by the total body fat mass, also depends on individual regional body fat distribution differences and subcutaneous adipose tissue ability to expand [72]. Assessment of body composition compartments like fat mass, fat-free mass and lean mass with metabolic derangements may be better indicators of cardiovascular (CVD) risk than BMI alone, especially cardiorespiratory fitness (CRF) [73]. The role of body composition in patients with HF-like reduced lean mass serve as surrogate marker for skeletal muscle mass, presence of sarcopenia, sarcopenic obesity, and cachexia, with reduced quality of life and worse prognosis [74].

Use of the term “obesity paradox” is scientifically and clinically imprecise, as it lumps together many findings whose relationships remain unproven. The term oversimplifies complex biological responses that can vary by disease and treatment [7]. This paradoxical benefit of a medically unfavorable phenotype is strong in overweight, and less in more severe or morbidly obese populations. In contrary, this phenomenon may represent a “lean paradox” where normal weight or underweight individuals may have a poorer prognosis with respect to CVD, as a result of a progressive catabolic state and lean mass loss [73].

Better characterization and understanding of the visceral/ectopic fat depots role and their biochemical activity, body mass composition, CRF, and effects of weight loss are needed [75]. Subsequent research could shed light on crucial questions and help optimize the approach to HF patients and achieve the best possible outcomes.
